# Body Mass Index and Clinical Outcomes in Adult COVID-19 Patients of Diverse Ethnicities

**DOI:** 10.3390/healthcare10122575

**Published:** 2022-12-19

**Authors:** Wael Hafez, Mahmoud Abdelshakor, Samy Kishk, Amr Gebril, Muneir Gador, Sana Osman, Hesham Mohamed Abuelsaoud, Ahmed Abdelrahman

**Affiliations:** 1 NMC Royal Hospital, 16th Street, Khalifa City, Abu Dhabi P.O. BOX 764659, United Arab Emirates; 2 The Medical Research Division, Department of Internal Medicine, The National Research Center, El Buhouth Street, Ad Doqi, Cairo 12622, Egypt; 3 The Internal Medicine Department, Faculty of Medicine, Zagazig University, Zagazig 44519, Egypt

**Keywords:** obesity, body mass index, COVID-19, pneumonia, severity, United Arab Emirates

## Abstract

(1) Background: Body mass index (BMI) was observed to affect COVID-19 outcomes; however, the complete spectrum of clinical outcomes concerning BMI remains unexplored. The current study aimed to investigate the correlation between BMI and the severity and mortality of COVID-19, as well as ICU admission, radiological findings, clinical presentation, and time to viral clearance. (2) Methods: This retrospective study included 1796 multiethnic patients with COVID-19 treated at NMC Royal Hospital, Abu Dhabi, UAE. (3) Results: COVID-19’s adjusted odds of severity increased by 3.7- and 21.5-fold in classes I and III, respectively (*p* = 0.001). The odds of mortality were not significantly different after adjustment for age, sex, and race. The adjusted odds of ICU admission increased significantly by 3-fold and non-significantly by 4-fold in obesity classes I and II, respectively. Pneumonia was significantly higher in patients who were overweight and class I, II, and III obese. Furthermore, class III obese patients had a greater risk of presenting with combined respiratory and gastrointestinal manifestations (*p* < 0.001). The median time to viral clearance with a BMI > 40 kg/m^2^ was moderately higher than that with a BMI < 40 kg/m^2^. (4) Conclusions: High BMI was associated with pneumonia, ICU admission, severity, and mortality due to COVID-19.

## 1. Introduction

Obesity has been recognized as a primary contributing factor to the severity and mortality of coronavirus disease 2019 (COVID-19) [1]. Many studies have also linked some other chronic diseases with COVID-19 severity [2,3,4,5].

Hypertension, being the most prevalent cardiovascular comorbidity in patients infected with COVID-19, was shown to tremendously increase the risk of hospitalization and death [6]. Coronary artery disease/cardiovascular disease (CAD/CVD) and chronic pulmonary disease were also both strongly correlated with disease severity [7]. On the other hand, patients with rheumatic diseases showed no increased risk of COVID-19 severity in comparison to other comorbidities [8].

A total of 51,633 COVID-19 patients in Mexico were the subject of a large study that reported that obese patients had increased odds of entering the intensive care unit (ICU), getting intubated, and being hospitalized [9]. Obesity was also identified as a risk contributor to death in the United Kingdom cohort research, which comprised 20,133 participants [10]. Moreover, a body mass index (BMI) study in New York was performed on 10,861 COVID-19 patients and showed that underweight, overweight, and obese patients were linked to poor outcomes in hospitalized individuals positive for COVID-19 [11].

In the Netherlands, 67 COVID-19 ICU-admitted patients were studied and classified into obese and nonobese groups. Unlike other reports, here, the authors found that a greater BMI is not associated with various immunological reactions, undesirable respiratory mechanics, or poor outcomes in patients requiring mechanical ventilation in the ICU who are positive for COVID-19 [12].

Although COVID-19 patients’ clinical results have been linked to obesity (including overweight (BMI 25.0 to <30) and three classes of obesity: class I (BMI = 30 to <35), class II (BMI = 35 to <40), and class III (BMI ≥ 40)) [13], the connection between the full scope of body physique based on BMI and outcomes of COVID-19 is still not fully understood. Additionally, there are scarce data regarding the link between BMI or obesity and COVID-19 susceptibility and outcomes in the multiethnic population of the United Arab Emirates (UAE). Recently, no association was reported between obesity and survival after COVID-19 in the UAE by Nair et al.; however, the correlation between obesity and other COVID-19 outcomes (such as pneumonia or time to viral clearance) was not examined by the authors [14]. Another study by Heialy et al. suggested that BMI alone was not related to poor COVID-19 outcomes among COVID-19 patients in the UAE, but the comorbidities present with a high BMI were linked to poor COVID-19 outcomes, including acute respiratory distress syndrome (ARDS) development, mechanical ventilation need, and mortality [15].

The current study aimed to investigate the correlation between BMI and the severity and mortality of COVID-19, ICU admission, radiological findings, clinical presentation, and time to viral clearance.

## 2. Materials and Methods

### 2.1. Ethical Considerations

This study was performed according to the Declaration of Helsinki. The Regional Research Ethics Committee, Department of Health, Abu Dhabi, UAE (DOH/CVDC/2021/1329), the NMC Central Scientific Committee (NMCHC/CSC/2011/0009), and the NMC Regional Ethics Committee (NMC/PREC/AUH/2021/0010) allowed the study. Identifiers for patients were not included in the data collection, with full privacy protection for patients. Due to the observational nature of the study, informed consent was not needed.

### 2.2. Study Design and Participants

A retrospective study was conducted utilizing COVID-19 patients’ medical records who received care at NMC Royal Hospital, Khalifa City, Abu Dhabi, UAE, from September 9 through 31 November 2020.

Incorporated patients tested positive for SARS-CoV-2 under aseptic conditions through the use of nasopharyngeal swab samples in a reverse transcriptase-polymerase chain reaction (RT-PCR). Patients participating in the study were 18 years old or above with different grades of COVID-19 severity according to the WHO classification (severe: severe to critical, and non-severe: mild to moderate). Demographic and clinical data, severity, ICU admission, mortality, and viral clearance time were extracted from electronic records.

### 2.3. Nasopharyngeal PCR Test of SARS-CoV-2

Infections with COVID-19 were identified in the laboratory using nasopharyngeal swab specimens and Solgent’s 2019-nCoV RT-PCR kit. The Thermal Cycler PCR (Bio-Rad, CA, USA) and CFX-96 plate reader ((Bio-Rad, Hercules, CA, USA) were utilized as directed by the manufacturer for RT-PCR analysis and viral detection. A positive result was that of a cycle threshold (C_T_) value greater than 40 in a sample. Positive results are indicative of the presence of SARS-CoV-2 RNA but must be clinically correlated with patient history and other diagnostic information to determine the infection status. They also do not rule out bacterial infection or coinfection with other viruses. Negative results must be combined with clinical observations, patient history, and epidemiological information to preclude SARS-CoV-2 infection [16]. The UAE’s national guidelines for the therapy and clinical management of COVID-19 were the basis for all patients’ testing protocols.

Testing was performed on each patient upon arrival to the clinic for outpatients or the emergency room, and patients with COVID-19 infection confirmed by RT-PCR were retested every five days, whereas patients without COVID-19 infection were retested after twenty-four hours. The initial negative RT-PCR test of two consecutive negative RT-PCRs determined the viral clearance date.

The number of days between the initial positive SARS-CoV-2 RT-PCR test result and the initial negative result of two consecutively negative RT-PCRs was utilized to determine how long it will take the virus to eradicate (viral clearance time).

### 2.4. Data Collection

Clinical and demographic information for each subject were gathered from the electronic medical records, laboratory and radiological findings, interventions for therapy, and disease consequences.

Retrospective analysis of clinical data was performed by searching the information from our cloud hospital system “INSTA”, including the severity of the condition, age, sex, race, any concurrent diseases, clinical symptoms, ICU admission, mortality, pneumonia, and time to viral clearance. The system also included the daily progress notes for the patients, physicians and nursing notes, patient progress every 4 h, medications ordered, and radiological data. The identifiable patient information was not used, and the study was conducted with patient privacy intact.

The clinical outcomes, including patient severity and mortality, were collected from the daily doctor notes, nursing notes, and other sheets, including patient progress every 4 h.

BMI is classified into underweight (<18.5), normal (18.5 to <25), overweight (25.0 to <30), and obese (30.0 or higher). Additionally, there are three classes of obesity: class I (BMI = 30 to <35), class II (BMI = 35 to <40), and class III (BMI ≥ 40) [13].

At the time of admission and throughout, baseline laboratory tests were performed, including inflammatory markers suggestive of a serious illness (platelet, lymphocyte count, D-dimer, lactate dehydrogenase (LDH), fibrinogen, C-reactive protein (CRP), and others). Chest X-ray and/or chest computed tomography (CT) were performed on patients on presentation, and some underwent a second chest X-ray and/or CT of the chest at varying intervals based on clinical evaluation.

The radiological data were available on the INSTA system for revision at any time. The worst significant findings were considered in the case of several X-ray films and/or chest CT, so the most significant and deteriorated films were used for the analysis.

Based on the UAE and the WHO guidelines for the severity scale of COVID-19, fever was defined as an axillary temperature > 37.3 °C. Mild cases were defined as confirmed symptomatic COVID-19 patients without viral pneumonia or hypoxia. Moderate cases were confirmed for COVID-19 patients clinically presenting with symptoms of pneumonia, including cough, dyspnea, fever, or fast breathing, and without signs of severe pneumonia (as SpO2 ≥ 93% in room air). Severe cases were the same as those of moderate disease in addition to a respiratory rate above 30 breaths/min, severe respiratory distress symptoms, or SpO2 < 93% in room air. The critical case was defined as ARDS developed within one week of a known clinical complication in addition to new or deteriorating respiratory symptoms. Clinical improvement measurement was performed according to the WHO ordinary scale for clinical improvement of COVID-19. The time needed for viral clearance (time to viral clearance) was measured as the amount of time elapsed between the initial positive and initial negative PCR results of two successive negative results [17].

After data collection, all data generated during the study, either in physical form or digital form, were anonymized and were confidentially stored with restricted access only to the study’s investigator. If required by any external representative or authority, access to the data was allowed and given to them by the investigator. Data storage was as per the NMC MRD policy.

### 2.5. Statistical Analysis

Following the gathering and validation of data, statistical analysis was performed using R Software version 3.5.2 (20 December 2018), “Eggshell Igloo,” Copyright (C) 2018 The R Foundation for Statistical Computing, Platform: i386-w64-mingw32/i386 (32-bit) “source”, Egypt. Normally distributed quantitative data are presented as the mean ± standard deviation (SD) and range. The median and interquartile range were used to illustrate the data when the normal distribution was invalidated. Frequency (n) and percentage (%) were used to display the qualitative data. A logistic regression model was performed to explore the association between BMI and disease outcomes among COVID-19 patients, including disease severity (severe, non-severe), ICU admission, mortality, and radiological findings. Additionally, the time to viral clearance in relation to BMI was evaluated using Kaplan-Meier survival analysis. The confidence interval (CI) was 95%, and a 5% error margin was approved. Thus, the significance of the *p*-value was as follows: *p* > 0.05: non-significant, and *p* < 0.05: significant.

## 3. Results

### 3.1. Sociodemographic and Clinical Characteristics of Participants

The study was performed on 1796 hospitalized COVID-19 patients. Males represented more than 85% of the study population. Nearly 80% of males had a BMI between 18.5 and 29.9 kg/m^2^ (normal and overweight), and approximately 19% had a BMI above 30 kg/m^2^ (obesity class I, obesity class II, and obesity class III). From the study population, South Asians represented 73.7%, East Asians represented 6.6%, Middle Eastern represented 15.0%, Black/Africans represented 2.9%, American/Alaska Native represented 0.5%, and white European represented 1.4%. More than 80% of American/Alaska Native, East Asians, and South Asians, more than 60% of Middle Eastern and White Europeans, and nearly 60% of Black/Africans were normal and overweight. Regarding comorbidities, more than 60% of hypertensive and diabetic patients and more than 50% of cardiovascular or chronic kidney disease patients were overweight and obese class I. Clinical and demographic details were compared in-depth and are shown in Table 1.

### 3.2. Logistics Regression Analysis of the Relationship between BMI and COVID-19 and Outcomes

**Figure 1:**
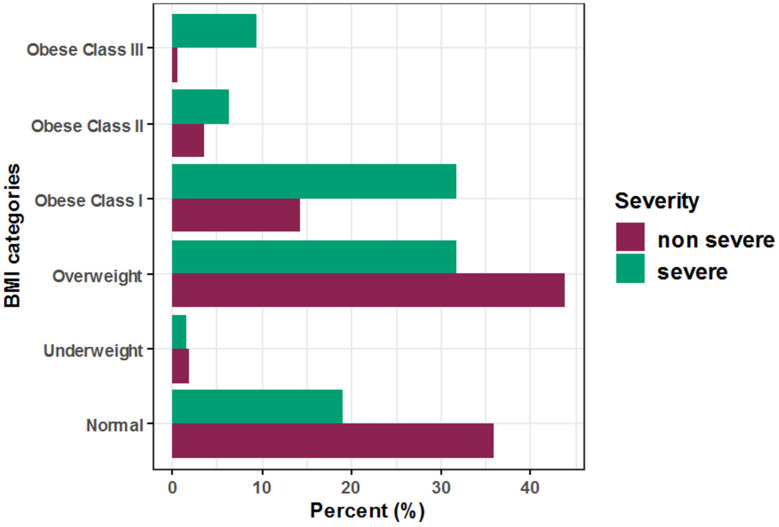
Association between BMI and disease severity among hospitalized COVID-19 patients.

The independent association between BMI and other COVID-19 patient disease consequences has been further studied. Regarding disease severity, the unadjusted odds increased significantly by approximately 4.2-, 3.3-, and 30.6-fold among obese COVID-19 patients in class I, class II, and class III, respectively, while after adjustment for patient age, sex, and race, the adjusted odds increased significantly by approximately 3.7- and 21.5-fold among obese COVID-19 patients in class I and class III, respectively, when compared to normal BMI patients (Unadj. OR = 4.20, 95% CI: (2.05–8.98), *p* < 0.001), (Unadj. OR = 3.30, 95% CI: (0.90–9.79), *p* = 0.044), (Unadj. OR = 30.65, 95% CI: (9.21–97.67), *p* < 0.001), (Adj. OR = 3.72, 95% CI: (1.73–8.31), *p* = 0.001), and (Adj. OR = 21.51, 95% CI: (5.16–84.23), *p* < 0.001). Additionally, for each one-year increase in COVID-19 patient age, the unadjusted and adjusted odds of severity increased significantly by approximately 13% for both (Unadj. OR = 1.13, 95% CI: (1.10–1.15), *p* < 0.001) and (Adj. OR = 1.13, 95% CI: (1.10–1.16), *p* < 0.001), respectively (Table 2, Figure 1).

**Figure 2:**
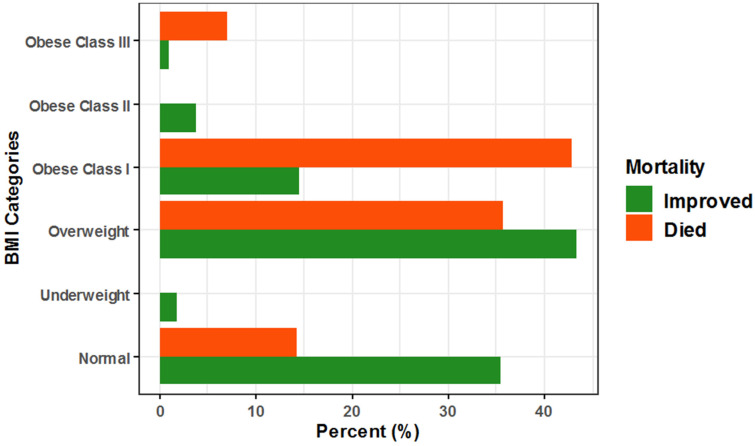
Association between BMI and disease mortality among hospitalized COVID-19 patients.

Regarding mortality, the unadjusted odds increased significantly by approximately 7.3- and 20.8-fold among obese COVID-19 patients in class I and class III, respectively, compared to normal BMI patients, and after adjustment for patient age, sex, and race, the adjusted odds of mortality increased, but non-significantly, by approximately 4.9- and 4.4-fold, respectively (Unadj. OR = 7.27, 95% CI: (1.66–49.86), *p* = 0.016), (Unadj. OR = 20.77, 95% CI: (0.94–228.64), *p* = 0.015), (Adj. OR = 4.94, 95% CI: (0.92–26.7), *p =* 0.063), and (Adj. OR = 4.44, 95% CI: (0.24–80.89), *p =* 0.314). Additionally, for each one-year increase in COVID-19 patient age, the unadjusted and adjusted odds of mortality increased significantly by approximately 17% for both (Unadj. OR = 1.17, 95% CI: (1.12–1.24), *p* < 0.001) and (Adj. OR= 1.17, 95% CI: (1.1–1.24), *p* < 0.001), respectively (Table 3, Figure 2).

**Figure 3:**
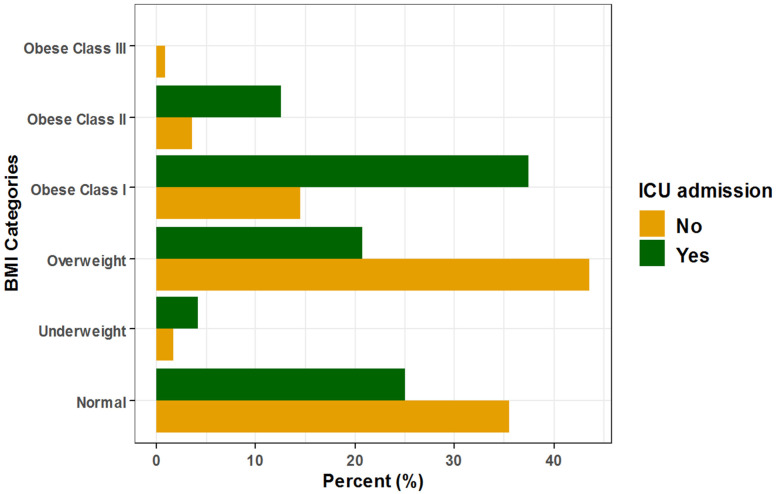
Association between BMI and ICU admission among hospitalized COVID-19 patients.

Regarding ICU admission, the unadjusted odds increased significantly by approximately 3.7- and 4.9-fold among class I and class II obese COVID-19 patients, respectively, compared to normal BMI patients, and after adjustment for patient age, sex, and race, the adjusted odds of ICU admission increased significantly by approximately 3-fold and non-significantly by approximately 4-fold, respectively (Unadj. OR = 3.66, 95% CI: (1.30–11.00), *p* = 0.015), (Unadj. OR = 4.91, 95% CI: (1.02–19.10), *p* = 0.027), (Adj. OR = 2.97, 95% CI: (1.00–8.83), *p =* 0.0496), and (Adj. OR = 3.96, 95% CI: (0.82–19.05), *p =* 0.086). Additionally, for each one-year increase in COVID-19 patient age, the unadjusted and adjusted odds of ICU admission increased significantly by approximately 11% and 13%, respectively (Unadj. OR = 1.11, 95% CI: (1.08–1.15), *p* < 0.001) and (Adj. OR = 1.13, 95% CI: (1.08–1.17), *p* < 0.001), respectively (Table 4, Figure 3).

**Figure 4:**
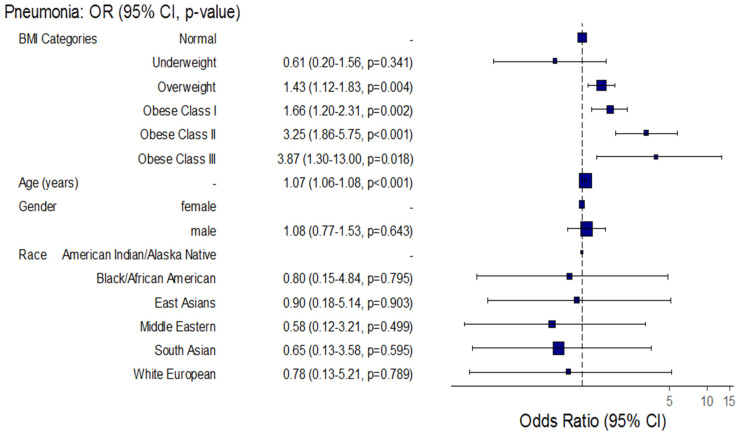
Forest plot for the association between BMI and the incidence of pneumonia among hospitalized COVID-19 patients.

Regarding pneumonia, the unadjusted odds increased significantly by approximately 57%, 97%, 3.6-fold, and 5.5-fold among overweight, obese class I, obese class II, and obese class III patients, respectively, compared to normal BMI patients, while after adjustment for patient age, sex, and race, the adjusted odds of pneumonia increased significantly by approximately 43%, 66%, 3.3-fold, and 3.9-fold, respectively (Unadj. OR = 1.57, 95% CI: (1.24–1.98), *p* < 0.001), (Unadj. OR = 1.97, 95% CI: (1.45–2.68), *p* < 0.001), (Unadj. OR= 3.58, 95% CI: (2.11–6.16), *p* < 0.001), (Unadj. OR= 5.54, 95% CI: (1.98–17.80), *p* = 0.002), (Adj. OR = 1.43, 95% CI: (1.12–1.83), *p* = 0.004), (Adj. OR = 1.66, 95% CI: (1.20–2.31), *p* = 0.002), (Adj. OR = 3.25, 95% CI: (1.86–5.75), *p* < 0.001), and (Adj. OR = 3.87, 95% CI: (1.30–13.00), *p* = 0.018). Additionally, for each one-year increase in COVID-19 patient age, the unadjusted and adjusted odds of pneumonia increased significantly by approximately 7% for both (Unadj. OR = 1.07, 95% CI: (1.06–1.09), *p* < 0.001) and (Adj. OR = 1.07, 95% CI: (1.06–1.08), *p* < 0.001), respectively (Table 5, Figure 4).

**Figure 5:**
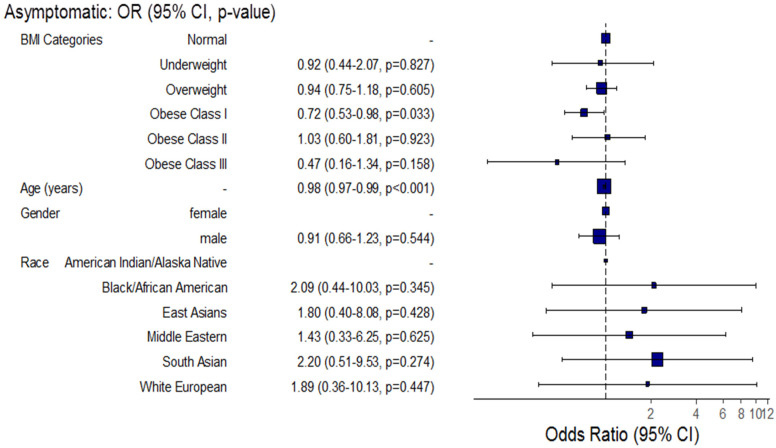
Forest plot for the association between BMI and asymptomatic hospitalized COVID-19 patients.

Regarding the main clinical presentation, the adjusted odds of asymptomatic COVID-19 cases decreased significantly by approximately 28% among obese class I COVID-19 patients compared to normal BMI patients (Adj. OR = 0.72, 95% CI: (0.53–0.98), *p =* 0.033) and decreased significantly by approximately 2% for each one-year increase in patient age (Adj. OR = 0.98, 95% CI: (0.97–0.99), *p* < 0.001) (Figure 5).

**Figure 6:**
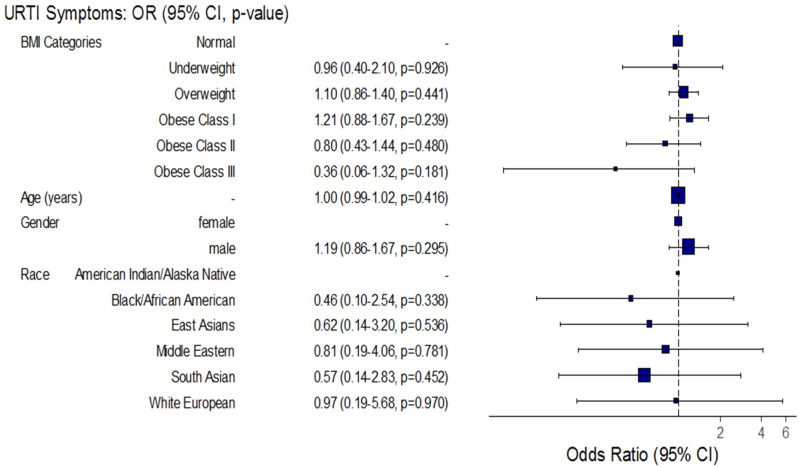
Forest plot for the association between BMI and the incidence of upper respiratory tract infection among hospitalized COVID-19 patients. BMI: body mass index; CI: confidence interval; URTI: upper respiratory tract infection; OR: odds ratio.

**Figure 7:**
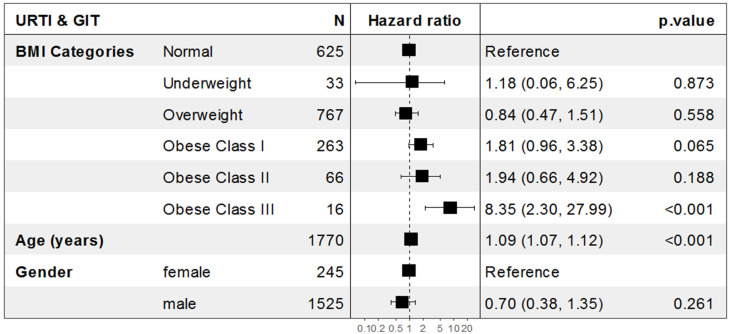
Forest plot for the association between BMI and the incidence of upper respiratory tract infection and gastrointestinal symptoms among hospitalized COVID-19 patients. BMI: body mass index; CI: confidence interval; URTI: upper respiratory tract infections; GIT: gastrointestinal tract infections; OR: odds ratio.

**Figure 8:**
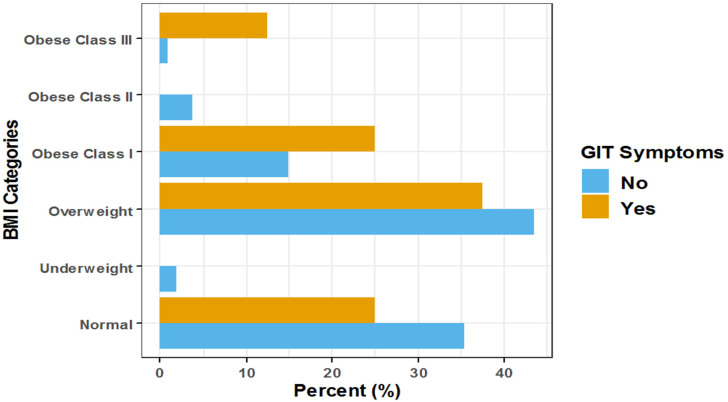
Forest plot for the association between BMI and the incidence of gastrointestinal symptoms among hospitalized COVID-19 patients. BMI: body mass index; GIT: gastrointestinal tract infection.

The adjusted odds of upper respiratory tract and gastrointestinal symptoms together increased significantly by approximately 8.4-fold among obese class III COVID-19 patients compared to normal BMI patients (Adj. OR = 8.35, 95% CI: (2.30–27.99), *p* < 0.001), and increased significantly by approximately 9% for each one-year increase in patient age (Adj. OR = 1.09, 95% CI: (1.07–1.12), *p* < 0.001) (Figure 6, Figure 7 and Figure 8).

### 3.3. Time to Viral Clearance in Patients with Various BMIs

**Figure 9:**
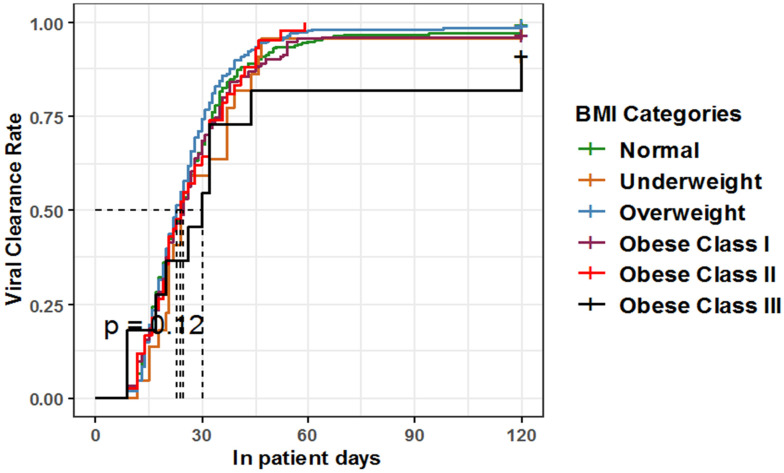
Kaplan-Meier estimate for time to viral clearance among hospitalized COVID-19 patients of different BMI categories.

The Kaplan-Meier curve showed a non-significant difference between different BMI categories regarding the median time for patients to reach viral clearance, showing overlapping confidence intervals (*p* = 0.1, log-rank = 8.7) (Table 6, Figure 9).

**Figure 10:**
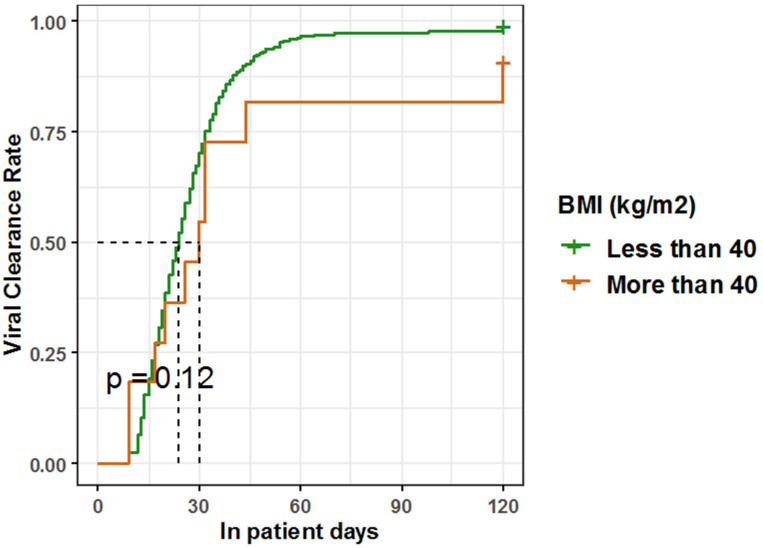
Kaplan-Meier estimate for time to viral clearance among hospitalized COVID-19 patients with a BMI of more than 40 versus less than 40.

Meanwhile, class III obese COVID-19 patients had a slightly delayed median time to viral clearance (30 days), so another Kaplan-Meier estimate was performed to compare the time to viral clearance in those classes of patients (BMI more than 40) with patients with a lower BMI, showing that the median time to viral clearance in patients with BMI > 40 is 30 days, which is moderately longer than the median time to viral clearance in patients with BMI < 40 (24 days), but without a statistically significant difference (*p* = 0.1, log-rank = 2.4). However, a clinically meaningful observation should still be taken into consideration while managing COVID-19 patients with a BMI more than 40 (Table 7, Figure 10).

## 4. Discussion

### 4.1. Significance of the Results

This retrospective study explored the correlation between BMI and clinical presentation of COVID-19, disease severity, mortality, ICU admission, radiological findings, clinical presentation, and time to viral clearance.

Several studies have focused on how a healthy lifestyle, which in turn maintains a normal BMI, can prevent COVID-19 severity. An example is a study by Aouissi et al., which showed that strong immunity due to healthy lifestyle measures was associated with a reduced probability of contracting the COVID-19 virus. They also demonstrated that eating healthy, sleeping well, and regular physical activity can better enhance multiple disease prevention and outcomes [18,19]. Another study on children by Scapaticci et al. discussed that being able to consume enough vitamins and minerals, which have antiviral properties and may lessen the intensity of symptoms, is essential for enhancing immune responses [20].

We found that there were increased odds of the severity of COVID-19 among all obesity classes, and the odds of severity remained significantly high among patients in class I and class III after adjusting for age, gender, and race. This finding was supported by the results of a well-powered study in the UK that verified an association between BMI and the possibility of hospitalization and death because of COVID-19 [21] and a case-control study using Swedish registers, which established obesity to be a dangerous element increasing the severity of COVID-19 disease [22]. Hegde et al. also reported that BMI > 23 kg/m^2^ is a significant risk factor for COVID-19 disease severity among the South Asian adult population [23]. Moreover, a new Mendelian randomization study found that having a high BMI increases one’s vulnerability to COVID-19 severity and susceptibility [24]. Another in vivo study found that COVID-19 could infect adipocytes and macrophages, leading to a dramatic inflammatory response, especially in adipose tissue adjacent to vital organs, which might explain the association between obesity and severe COVID-19 outcomes [25].

In a study of a 10,544 COVID-19 population, individuals with a BMI ranging from 30 to 40 kg/m^2^ had a greater risk of being hospitalized and clinical worsening in comparison to others with a BMI beneath 30 kg/m^2^ [26]. In a different study exploring infection with COVID-19, obesity alongside age ≥ 52 years was significantly associated with illness severity [27]. An investigation by Cai et al. [28] showed that increased disease intensity was linked with male sex. Likewise, Chiumello et al. [29] discovered a strong relationship between male sex and acute respiratory distress in overweight/obese patients.

Here, the odds of mortality significantly increased among patients in obesity classes I and III (*p* = 0.016 and 0.015, respectively). The difference in BMI of patients had a non-significant impact on the rate of either mortality or improvement of cases after adjustment. Additionally, the odds of ICU admission increased by approximately 3.7- and 4.9-fold among obese COVID-19 patients in class I and class II, respectively, before adjustment. After adjustment, it increased significantly by approximately 3-fold and non-significantly by approximately 4-fold among obese COVID-19 patients in class I and class II, respectively. These findings are consistent with those of Huang et al., who reported that a high BMI was significantly associated with increased odds of disease severity, mortality, hospitalization, ICU admission, and the need for mechanical ventilation [1].

A study including 100 individuals with COVID-19 reported that pneumonia and obesity did not indicate increased mortality [30]. In contrast to our findings, a study that included 9347 veterans observed a statistically higher COVID-19 mortality and ICU admission risk related to increased BMI (*p* < 0.05) [31]. Additionally, in a case-control study assessing the impact of BMI on COVID-19 mortality with a large sample size, the pooled results showed that patients with a BMI of 30 kg/m^2^ had a considerably greater risk of COVID-19 mortality, according to the random effects model (*p* < 0.001) [32]. In addition, a similar retrospective observational study of 125 medical records from HUSF indicated that obesity participates in metabolic changes and increased mortality in SARS-CoV-2-infected patients [33]. A recent meta-analysis showed that there was a linear dose-response association between BMI and severity and mortality due to COVID-19. Additionally, BMI ≥ 30 kg/m^2^ was linked with a significantly elevated risk of critical disease outcomes and in-hospital mortality [34].

In the current study, high odds of pneumonia were significantly associated with patients who were overweight, class I obese, class II obese, and class III obese before and after adjustment. Likewise, an exploratory study of 39 of 165 individuals with a COVID-19 PCR-based diagnosis reported an association of overweight/obesity with pneumonia signs in COVID-19 patients (OR = 2.68, CI: 1.29–5.59, *p =* 0.008) [35]. Obesity hypoventilation can be a reason for worsening hypoxemia in COVID-19 pneumonia. In addition, obesity could aggravate the cytokine release that occurs during COVID-19 pneumonia, which could lead to multiple organ failure and acute respiratory distress syndrome [36,37].

We observed that the odds of being asymptomatic were lower among patients in obesity class I. In contrast, the odds of GIT and URTI significantly increased among patients in obesity class III. Nevertheless, a slight increase in the median time needed to reach viral clearance was shown in class III obese patients (30 days), and further research may be necessary to evaluate the time to viral clearance in COVID-19 patients with BMI > 40 kg/m^2^ compared with that of patients with BMI < 40 kg/m^2^. The median time to reach viral clearance in COVID-19 patients with a BMI of more than 40 kg/m^2^ was moderately higher than that in patients with a BMI of less than 40 kg/m^2^. The *p*-value (>0.05) shows this difference to be non-significant. However, this difference could still be considered a clinically meaningful observation that should be considered while managing COVID-19 patients with a BMI value exceeding 40 kg/m^2^. Obesity-prompted insulin resistance may decrease efficient viral clearance and induce organ injury in severe COVID-19 progression [34]. Additionally, our findings were supported by a study of 100 consecutive COVID-19 pneumonia patients, which showed that obesity is related to a more severe respiratory presentation of COVID-19, delayed viral clearance, and prolonged hospital stay [38].

Importantly, we found that for every one-year increase in the age of COVID-19 patients, there was an increase in severe disease outcomes, pneumonia, ICU admission, and mortality, which is consistent with Palaiodimos et al.’s findings [39]. This could be explained by elevated fat mass in relation to muscle mass, increased prevalence of comorbidities, decreased cardiorespiratory reserve, and immune senescence [40].

Furthermore, a review of elderly patients to determine the association between elderly obesity and COVID-19 prognosis detected that dynapenic or sarcopenic obesity could be connected to poor prognosis and a higher risk of COVID-19 complications [41].

All these findings lead us to believe that better weight control can impact COVID-19 patients’ health and disease prognosis. In addition, patients with comorbid conditions are advised to use all precautionary measures possible to prevent them from being infected with COVID-19 so they can avoid severe and unpleasant disease outcomes.

### 4.2. Strengths and Limitations

Owing to the observational design of this study, the possibility of unquantified residual confounding exists. Measured rates might be affected by many other elements, including socioeconomic differences, smoking, advance directives, healthcare decisions, health insurance access, public transportation dependence, and overcrowding. In addition, the lack of an age factor is considered a limitation of our study. One final limitation is the small sample size in class II and III obese patients in comparison to other weight groups. Additionally, South Asians are dominant over all other races.

Despite these limitations, this study also provides several noteworthy advantages: It showed an association between COVID-19 prognosis and obesity in a large sample size with detailed BMI data. Furthermore, the study was conducted on a multiethnic population in the UAE.

## 5. Conclusions

This research on COVID-19 patients revealed that a high BMI was associated with significantly increased severity of COVID-19 outcomes. The odds of pneumonia increased with increasing BMI. However, BMI was not strongly associated with mortality or ICU admission. Class I obesity was more prone to be asymptomatic, while class III had higher odds of suffering from both URTI and GIT symptoms. Additionally, the time to viral clearance was increased by the increase in BMI level, although the difference was not statistically significant.

Future studies should examine other risk factors and comorbidities that endanger COVID-19 patients to aid physicians in making better decisions. We also advise exploring the effect of BMI while accounting for different ethnicities. All COVID-19 patients should have their BMI measured, and those who are obese should be given special attention. In our opinion, this study supports the theory that obesity is a contributing factor to COVID-19 complications and that healthcare providers ought to consider this when planning COVID-19 prevention and management strategies.

## Figures and Tables

**Table 1:** Baseline demographic characteristics based on BMI category.

			BMI Categories						*p*-Value
		Total	Underweight (<18.5) N = 33 (1.9%)	Normal (18.5–24.9) N = 625 (35.3%)	Overweight (25–29.9) N = 767 (43.3%)	Obese Class I (30–34.9) N = 263 (14.9%)	Obese Class II (35–39.9) N = 66 (3.7%)	Obese Class III (≥ 40) N = 16 (0.9%)	
**Age (years)**	**Mean (SD)**	36.7 (9.6)	31.5 (7.6)	35.0 (9.7)	37.2 (9.2)	38.7 (9.7)	39.0 (9.7)	43.9 (11.4)	<0.001
**Gender**	**Female**	245 (13.8)	11 (4.5)	90 (36.7)	88 (35.9)	39 (15.9)	10 (4.1)	7 (2.9)	0.002
	**Male**	1525 (86.2)	22 (1.4)	535 (35.1)	679 (44.5)	224 (14.7)	56 (3.7)	9 (0.6)	
**HTN**	**No**	1729 (97.7)	33 (1.9)	616 (35.6)	757 (43.8)	247 (14.3)	63 (3.6)	13 (0.8)	<0.001
	**Yes**	41 (2.3)	0 (0.0)	9 (22.0)	10 (24.4)	16 (39.0)	3 (7.3)	3 (7.3)	
**DM**	**No**	1722 (97.3)	33 (1.9)	616 (35.8)	751 (43.6)	250 (14.5)	60 (3.5)	12 (0.7)	<0.001
	**Yes**	48 (2.7)	0 (0.0)	9 (18.8)	16 (33.3)	13 (27.1)	6 (12.5)	4 (8.3)	
**CVS/CKD**	**No**	1757 (99.3)	33 (1.9)	622 (35.4)	763 (43.4)	260 (14.8)	65 (3.7)	14 (0.8)	0.013
	**Yes**	13 (0.7)	0 (0.0)	3 (23.1)	4 (30.8)	3 (23.1)	1 (7.7)	2 (15.4)	
**Race**	**American Indian/Alaska Native**	8 (0.5)	0 (0.0)	3 (37.5)	4 (50.0)	1 (12.5)	0 (0.0)	0 (0.0)	<0.001
	**Black/African American**	51 (2.9)	2 (3.9)	13 (25.5)	17 (33.3)	12 (23.5)	6 (11.8)	1 (2.0)	
	**East Asians**	117 (6.6)	4 (3.4)	41 (35.0)	56 (47.9)	13 (11.1)	2 (1.7)	1 (0.9)	
	**Middle Eastern**	266 (15.0)	4 (1.5)	71 (26.7)	99 (37.2)	57 (21.4)	27 (10.2)	8 (3.0)	
	**South Asian**	1304 (73.7)	23 (1.8)	492 (37.7)	581 (44.6)	173 (13.3)	29 (2.2)	6 (0.5)	
	**White European**	24 (1.4)	0 (0.0)	5 (20.8)	10 (41.7)	7 (29.2)	2 (8.3)	0 (0.0)	

BMI: body mass index; CI: confidence interval; HTN: hypertension; DM: diabetes mellitus; CVD/CKD: cardiovascular diseases/chronic kidney diseases.

**Table 2:** Logistic regression analysis of the association between BMI and COVID-19 severity.

Risk Factors		COVID-19 Severity	
		OR (Univariable)	OR (Multivariable)
**BMI categories**	Normal	-	-
	Underweight	1.60 (0.09–8.49, *p* = 0.658)	2.82 (0.14–17.16, *p* = 0.349)
	Overweight	1.37 (0.67–2.90, *p* = 0.396)	1.13 (0.54–2.48, *p* = 0.745)
	Obese Class I	4.20 (2.05–8.98, *p* < 0.001)	3.72 (1.73–8.31, *p* = 0.001)
	Obese Class II	3.30 (0.90–9.79, *p* = 0.044)	2.65 (0.64–9.07, *p* = 0.142)
	Obese Class III	30.65 (9.21–97.67, *p* < 0.001)	21.51 (5.16–84.23, *p* < 0.001)
**Age (years)**	Mean (SD)	1.13 (1.10–1.15, *p* < 0.001)	1.13 (1.10–1.16, *p* < 0.001)
**Gender**	Female	-	-
	Male	0.75 (0.40–1.53, *p* = 0.396)	0.90 (0.41–2.18, *p* = 0.808)
**Race**	American Indian/Alaska Native	-	-
	Black/African American	2,659,050.77 (0.00-NA, *p* = 0.992)	1,287,271.01 (0.00-NA, *p* = 0.991)
	East Asians	2,279,186.37 (0.00-NA, *p* = 0.992)	3,515,161.02 (0.00-NA, *p* = 0.990)
	Middle Eastern	2,858,742.33 (0.00-NA, *p* = 0.992)	1,230,728.79 (0.00-NA, *p* = 0.991)
	South Asian	1,224,073.14 (0.00-NA, *p* = 0.992)	1,597,262.47 (0.00-NA, *p* = 0.991)
	White European	1.00 (0.00-Inf, *p* = 1.000)	0.42 (0.00-Inf, *p* = 1.000)

BMI: body mass index; CI: confidence interval; ICU: intensive care unit; URTI: upper respiratory tract infections; GIT: gastrointestinal tract infection. The parenthesis after “n” indicates the percentage.

**Table 3:** Relationship between patient BMI and mortality due to COVID-19.

Risk Factors		Mortality	
		OR (Univariable)	OR (Multivariable)
**BMI categories**	Normal	-	-
	Underweight	0.00 (NA-Inf, *p* = 0.994)	0.00 (0.0–Inf, *p* = 0.997)
	Overweight	2.04 (0.44–14.31, *p* = 0.394)	1.37 (0.24–7.88, *p* = 0.728)
	Obese Class I	7.27 (1.66–49.86, *p* = 0.016)	4.94 (0.92–26.7, *p* = 0.063)
	Obese Class II	0.00 (NA-Inf, *p* = 0.992)	0.00 (0.0–Inf, *p* = 0.996)
	Obese Class III	20.77 (0.94–228.64, *p* = 0.015)	4.44 (0.24–80.89, *p* = 0.314)
**Age (years)**	Mean (SD)	1.17 (1.12–1.24, *p* < 0.001)	1.17 (1.1–1.24, *p* < 0.001)
**Gender**	Female	-	-
	Male	0.59 (0.18–2.60, *p* = 0.413)	0.55 (0.1–2.9, *p* = 0.477)
**Race**	American Indian/Alaska Native	-	-
	Black/African American	34,878,997.12 (0.00-NA, *p* = 0.998)	53,549,043.87 (0.0–Inf, *p* = 0.999)
	East Asians	1.00 (NA-Inf, *p* = 1.000)	2.11 (0.00–Inf, *p =* 1.000)
	Middle Eastern	16,123,309.99 (0.00-NA, *p =* 0.998)	12,997,425.08 (0.0–Inf, *p* = 0.999)
	South Asian	4,545,401.22 (0.00-NA, *p =* 0.998)	17,456,381.76 (0.0–Inf, *p* = 0.999)
	White European	1.00 (NA-Inf, *p* = 1.000)	0.34 (0.00–Inf, *p* = 1.000)

**Table 4:** Relationship between patient BMI and ICU admission among COVID-19 patients.

Risk Factors		ICU Admission	
		OR (Univariable)	OR (Multivariable)
**BMI categories**	Normal	-	-
	Underweight	3.22 (0.17–19.66, *p* = 0.285)	5.59 (0.57–54.46, *p =* 0.138)
	Overweight	0.68 (0.19–2.26, *p* = 0.521)	0.51 (0.15–1.76, *p =* 0.288)
	Obese Class I	3.66 (1.30–11.00, *p* = 0.015)	2.97 (1.00–8.83, *p =* 0.0496)
	Obese Class II	4.91 (1.02–19.10, *p* = 0.027)	3.96 (0.82–19.05, *p =* 0.086)
	Obese Class III	0.00 (NA-Inf, *p* = 0.990)	0.00 (0.0–Inf, *p =* 0.992)
**Age (years)**	Mean (SD)	1.11 (1.08–1.15, *p* < 0.001)	1.13 (1.08–1.17, *p* < 0.001)
**Gender**	Female	-	-
	Male	1.12 (0.38–4.79, *p* = 0.850)	0.82 (0.22–3.12, *p =* 0.772)
**Race**	American Indian/Alaska Native	-	-
	Black/African American	7,228,049.56 (0.00-NA, *p* = 0.995)	3,906,650 (0.00–Inf, *p =* 0.994)
	East Asians	988,451.22 (0.00-NA, *p* = 0.995)	1,422,229 (0.00–Inf, *p =* 0.995)
	Middle Eastern	1,739,079.59 (0.00-NA, *p* = 0.995)	687,170.6 (0.00–Inf, *p =* 0.995)
	South Asian	1,415,746.51 (0.00-NA, *p* = 0.995)	1,756,078 (0.00–Inf, *p =* 0.994)
	White European	1.00 (0.00-Inf, *p* = 1.000)	0.43 (0.00–Inf, *p =* 1.000)

**Table 5:** Relationship between patient BMI and the radiological findings of COVID-19.

Risk Factors		Radiological Finding: Pneumonia	
		OR (Univariable)	OR (Multivariable)
**BMI categories**	Normal	-	-
	Underweight	0.50 (0.17–1.23, *p* = 0.169)	0.61 (0.20–1.56, *p* = 0.341)
	Overweight	1.57 (1.24–1.98, *p* < 0.001)	1.43 (1.12–1.83, *p* = 0.004)
	Obese Class I	1.97 (1.45–2.68, *p* < 0.001)	1.66 (1.20–2.31, *p* = 0.002)
	Obese Class II	3.58 (2.11–6.16, *p* < 0.001)	3.25 (1.86–5.75, *p* < 0.001)
	Obese Class III	5.54 (1.98–17.80, *p* = 0.002)	3.87 (1.30–13.00, *p* = 0.018)
**Age (years)**	Mean (SD)	1.07 (1.06–1.09, *p* < 0.001)	1.07 (1.06–1.08, *p* < 0.001)
**Gender**	Female	-	-
	Male	1.14 (0.85–1.55, *p* = 0.393)	1.08 (0.77–1.53, *p* = 0.643)
**Race**	American Indian/Alaska Native	-	-
	Black/African American	0.95 (0.19–5.28, *p* = 0.952)	0.80 (0.15–4.84, *p* = 0.795)
	East Asians	0.98 (0.21–5.20, *p* = 0.983)	0.90 (0.18–5.14, *p* = 0.903)
	Middle Eastern	0.84 (0.18–4.32, *p* = 0.817)	0.58 (0.12–3.21, *p* = 0.499)
	South Asian	0.71 (0.16–3.63, *p* = 0.658)	0.65 (0.13–3.58, *p* = 0.595)
	White European	1.22 (0.22–7.40, *p* = 0.818)	0.78 (0.13–5.21, *p* = 0.789)

**Table 6:** Time to viral clearance in relation to BMI category of COVID-19 patients.

BMI	Median Time to Clearance (Days)	95% CI (Days)	*p*-Value	Log-Rank
**Normal**	24	23–26	0.1	8.7
**Underweight**	24	21–37		
**Overweight**	23	22–25		
**Obese Class I**	25	22–27		
**Obese Class II**	24	21–32		
**Obese Class III**	30	20–NA		

BMI: body mass index; CI: confidence interval.

**Table 7:** Time to viral clearance in relation to patients’ BMI of more than 40 versus less than 40.

BMI	Median Time to Clearance (Days)	95% CI (Days)	*p*-Value	Log-Rank
**Less than 40**	24	23–25	0.1	2.4
**More than 40**	30	20–NA		

BMI: body mass index; CI: confidence interval.

## Data Availability

Data are available upon request from the first and corresponding author.
